# Target Deletion of the Cytoskeleton-Associated Protein Palladin Does Not Impair Neurite Outgrowth in Mice

**DOI:** 10.1371/journal.pone.0006916

**Published:** 2009-09-04

**Authors:** Run-Zhe Shu, Feng Zhang, Xue-Song Liu, Chun-Liang Li, Long Wang, Yi-Lin Tai, Xiao-Lin Wu, Xue Yang, Xiao-Dong Liao, Ying Jin, Ming-Min Gu, Lei Huang, Xiao-Fen Pang, Zhu-Gang Wang

**Affiliations:** 1 Key Laboratory of Stem Cell Biology, Institute of Health Sciences, Shanghai Institutes for Biological Sciences of Chinese Academy of Sciences and Shanghai Jiao Tong University School of Medicine (SJTUSM), Shanghai, China; 2 Model Organism Division, Department of Medical Genetics, E-Institutes of Shanghai Universities, Shanghai Jiao Tong University School of Medicine (SJTUSM), Shanghai, China; 3 Shanghai Research Center for Model Organisms, Shanghai, China; 4 Institute of Neurosciences, Shanghai Institutes for Biological Sciences, Chinese Academy of Sciences, Shanghai, China; 5 Department of Geratology, Rui-Jin Hospital, Shanghai Jiao Tong University School of Medicine (SJTUSM), Shanghai, China; 6 Graduate School of Chinese Academy of Sciences, Shanghai, China; New York State Institute for Basic Research, United States of America

## Abstract

Palladin is an actin cytoskeleton–associated protein which is crucial for cell morphogenesis and motility. Previous studies have shown that palladin is localized to the axonal growth cone in neurons and may play an important role in axonal extension. Previously, we have generated palladin knockout mice which display cranial neural tube closure defect and embryonic lethality before embryonic day 15.5 (E15.5). To further study the role of palladin in the developing nervous system, we examined the innervation of palladin-deficient mouse embryos since the 200 kd, 140 kd, 90–92 kd and 50 kd palladin isoforms were undetectable in the mutant mouse embryo brain. Contrary to the results of previous studies, we found no inhibition of the axonal extension in palladin-deficient mouse embryos. The cortical neurons derived from palladin-deficient mice also showed no significant difference in neurite outgrowth as compared with those from wild-type mice. Moreover, no difference was found in neurite outgrowth of neural stem cell derived-neurons between palladin-deficient mice and wild-type mice. In conclusion, these results suggest that palladin is dispensable for normal neurite outgrowth in mice.

## Introduction

Neurite outgrowth in immature neurons is an essential differentiation step during embryonic and adult neurogenesis. Neurite outgrowth, which is driven by actin-based motility of the growth cone, is critical for the establishment of the neuronal network. Both the initiation and the extension of neurites require organized actin polymerization to push the cell membrane forward. Thus, this actin-dependent process is closely associated with many actin cytoskeleton–associated proteins, such as Ena/VASP proteins, α-actinin, and profilin [1], [2].

Palladin is a widely expressed actin cytoskeleton-associated protein and is localized at stress fibers, focal adhesions, and other actin-based structures. In mouse embryos palladin is highly and ubiquitously expressed after E9.5 [3], [4]. It has been proven that palladin binds to F-actin and other actin-associated proteins such as VASP, α-actinin, profilin, Eps8, and ezrin [5]–[9]. Previous *in vitro* studies have shown that palladin is localized to the axonal growth cone in cortical neurons and may play an important role in axonal extension [10].

Mouse palladin gene is composed of at least 25 exons. So far, it has been proved that palladin gene has 3 alternative promoters and gives rise to at least 4 palladin protein isoforms including the 200 kd, 140 kd, 90–92 kd and 50 kd isoforms [11], [12]. To study the function of palladin *in vivo*, we generated palladin gene knockout mice through homologous recombination. In our knockout mouse model, the region between exon 16 and part of exon 18 was deleted, which is part of the common C-terminal region for the isoforms mentioned above, and 90–92 kd and 50 kd isoforms were confirmed ablated previously by Western blot analysis [4]. In addition, we found that the 200 kd, 140 kd and 90–92 kd palladin isoforms were not expressed in the palladin-deficient mouse brian.

Using this mouse model, we found that the disruption of palladin in mice results in cranial neural tube closure defect (NTD), herniation of liver and intestine, definitive erythropoiesis defects and embryonic lethality before E15.5, suggesting a crucial role for palladin during mouse embryogenesis [4], [13]. However, our results obtained from the palladin-deficient mouse embryos revealed no apparent phenotype with respect to neurite outgrowth: the peripheral nerve elongation was not stunted as compared to that in wild-type embryos. Moreover, *in vitro* quantitative neurite outgrowth assays using either palladin-deficient primary cortical neurons or neural stem cell (NSC)-derived neurons displayed no significant defect in neurite outgrowth and elongation in comparison with wild-type cells. These results indicate that target deletion of palladin does not impair neurite outgrowth in mice.

## Results

### Palladin knockout mice do not express the palladin isoforms sharing the common C-terminus in the E13.5 brain

**Figure 1:**
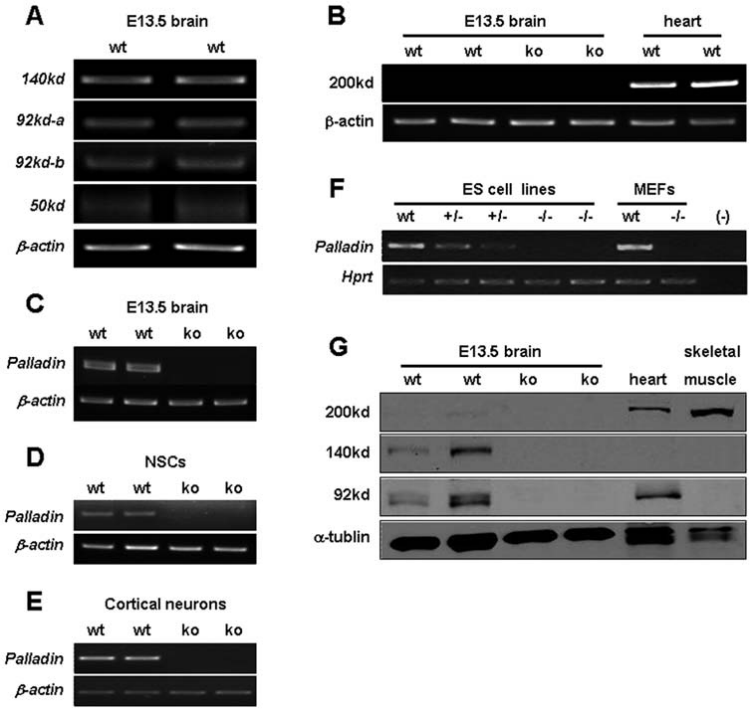
Detection of palladin isoforms in wild-type and palladin knockout mice. All transcripts of the isoforms as indicated except the 200 kd isoform were detected in E13.5 mouse brain by RT-PCR using isoform-specific primers (A and B). Using the primers aligned within the common C-terminal region, these transcripts were also detected in wild-type but not in palladin-deficient E13.5 brain (C), neural stem cells (D) and cortical neurons (E), as well as ES cells and MEFs (F). β-actin or Hprt is shown as internal control. Absence of 200 kd isoform in wild-type E13.5 brain and the other two isoforms (140 kd and 92 kd) in palladin-deficient brain was further confirmed by Western blot analysis using a C-terminal palladin antibody (G). α-tublin is shown as a loading control.

Mouse palladin gene gives rise to at least 4 palladin protein isoforms including the 200 kd, 140 kd, 90–92 kd and 50 kd isoforms, and these 4 isoforms shares the common C-terminus [11], [12]. In our knockout mouse model, the 90–92 kd and 50 kd isoforms were confirmed ablated previously by Western blot analysis [4]. To determine the role of each palladin isoform in the developing nervous system, we first tested whether palladin isoforms mentioned above are expressed in E13.5 mouse brain. Many lines of evidence have proved that the 200 kd isoform is not expressed in mouse brain [10]–[12]. Consistent with previous report, our data showed that the transcripts of these isoforms except the 200 kd isoform were detected in E13.5 mouse brain by RT-PCR using isoform-specific primers (Figure 1A and B). Moreover, using the primers aligned within the common C-terminal region, these transcripts were also detected in wild-type but not in palladin-deficient E13.5 brain (Figure 1C), NSCs (Figure 1D) and cortical neurons (Figure 1E), as well as ES cells and mouse embryonic fibroblasts (MEFs) (Figure 1F). The absence of 200 kd isoform in wild-type E13.5 brain and the other two isoforms (140 kd and 92 kd) in palladin-deficient brain was further confirmed by Western blot analysis (Figure 1G). These data indicate that the palladin isoforms detected were all missing in the E13.5 palladin-deficient mouse brain.

### Peripheral nerves are not significantly stunted in palladin-deficient embryos

**Figure 2:**
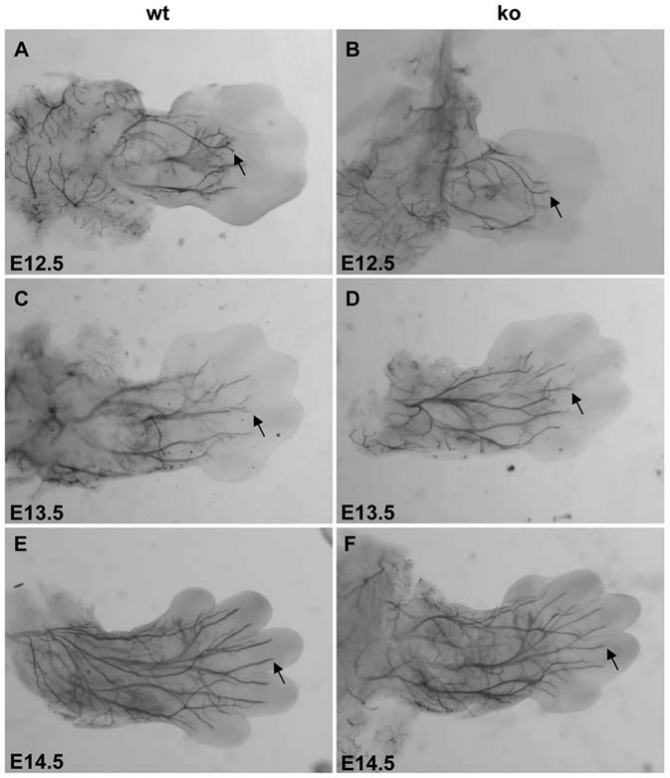
Neurofilament whole-mount immunohistochemistry of the developing limbs in mouse embryos. Shown is a dorsal view of the forelimbs of wild-type (wt, A, C, E) and palladin-deficient embryos (ko, B, D, F) at ages E12.5 (A, B), E13.5 (C, D), and E14.5 (E, F). Arrows indicate immunoreactive axons projecting into the forelimbs (magnification, 40×).

Palladin is an actin-associated protein which is proposed to be involved in the development of the nervous system. To determine whether palladin affects the morphogenesis and axonal growth of neurons during development, we examined neurofilament (NF) staining of the peripheral nerves between E12.5 and E14.5 in wild-type and palladin-deficient embryos. Whole-mount immunohistochemistry using antibodies against NF allowed us to visualize the morphology of axons in the limbs and head as described previously [14], [15]. Unexpectedly, palladin-deficient mice did not exhibit deficiencies of innervation to the forelimbs as they developed from E12.5 to E14.5 (n≥3). NF immunopositive axons in the forelimb of palladin-deficient embryos extended as far into the limb and branched as extensively as those in wild-type embryos (Figure 2). These data demonstrate that axon growth and morphology of dorsal root ganglia (DRG) neurons innervating the limbs is normal in the absence of palladin.

**Figure 3:**
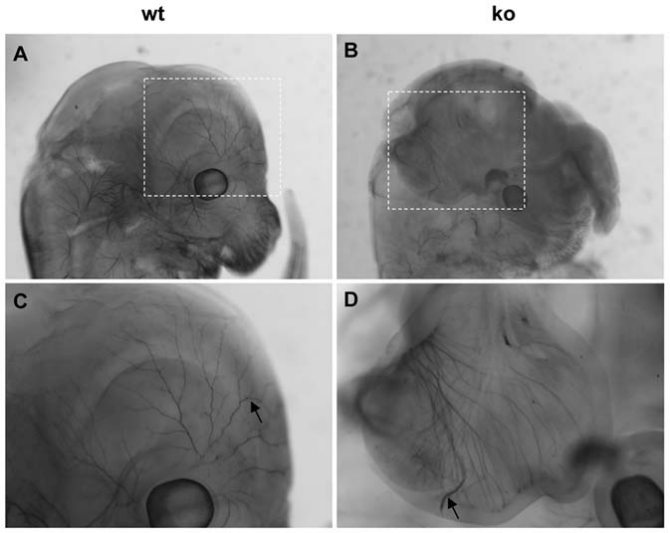
Neurofilament whole-mount immunohistochemistry of the skin from heads of mouse embryos. Shown are sagittal views of the heads of wild-type (wt, A, C) and palladin-deficient embryos (ko, B, D) at age E13.5. Arrows indicate immunoreactive axons (magnification, 20×).

In the E13.5 wild-type embryos, innervation to the skin above the eye originated from the trigeminal ganglion (Figure 3A and C). In E13.5, palladin-deficient embryos displayed exencephaly derived from neural tube closure defects [4]. The E13.5 palladin-deficient mouse embryo neuroepithelium could not fuse together to form normal cephalic ventricle structure as is present in the wild-type embryo (Supplementary Figure S1). As a result, the skin structure and innervation were both perturbed in the heads of palladin-deficient embryos. However, the neurite processes beneath the neuroepithelium were not stunted. Instead, they were extended in typical fashion (Figure 3B and D). In addition, innervation of the vibrissae and spinal cord was also normal in the palladin-deficient embryos (data not shown). Taken together, these results clearly demonstrate that palladin is not essential for normal axon outgrowth in mouse embryos.

### Neurite outgrowth is not inhibited in palladin-deficient cortical neurons

**Figure 4:**
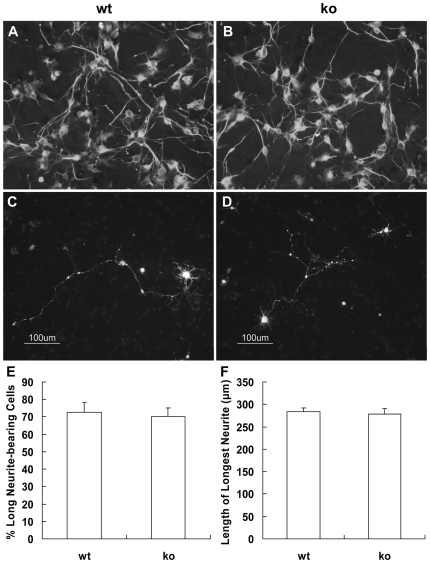
Target deletion of palladin does not inhibit neurite extension from primary cortical neurons in culture. Fluorescence immunostaining of Tuj1 in wild-type (wt, A) and palladin-deficient (ko, B) primary cortical neurons (4DIV). Representative examples of GFP transfected wt (C) and ko (D) neurons (4DIV) are shown. Quantification of neurite outgrowth based upon the GFP positive neurons described above. Cells with neurites of longer than two soma diameters were scored as long neurite-bearing cells. The percentage of long neurite-bearing cells is not decreased in palladin-deficient neurons (E). The length of the longest neurite is not significantly reduced in palladin-deficient neurons (F). Results are expressed as the mean±SEM (n = 3).

Since the nerves were not stunted in the palladin-deficient embryos, we then tested whether neurite outgrowth was affected *in vitro*. The palladin-deficient embryos died before E15.5, so we used cortical neurons from E14.5 embryos. Representative images show that there was no apparent difference in neurite length between wild-type and palladin-deficient neurons, both of which were able to extend long neurites (Figure 4A and B). In order to analyze neurite outgrowth quantitatively, we transfected cortical neurons with GFP to mark the neurites (Figure 4C and D). Among GFP positive neurons, 72.5±5.8% of wild-type neurons had neurites longer than two soma diameters, and 70.0±5.2% showed this response in the case of palladin-deficient neurons (*P* = 0.763836) (Figure 4E). In addition, the length of the longest neurite among wild-type neurons was 283.6±8.9 µm, and that of palladin-deficient neurons was 278.8±11.7 µm (*P* = 0.757526) (Figure 4F). These data show that neurite growth of cortical neurons is not significantly inhibited in the absence of palladin.

### Neurite outgrowth is not impaired in palladin-deficient neural stem cell-derived neurons

**Figure 5:**
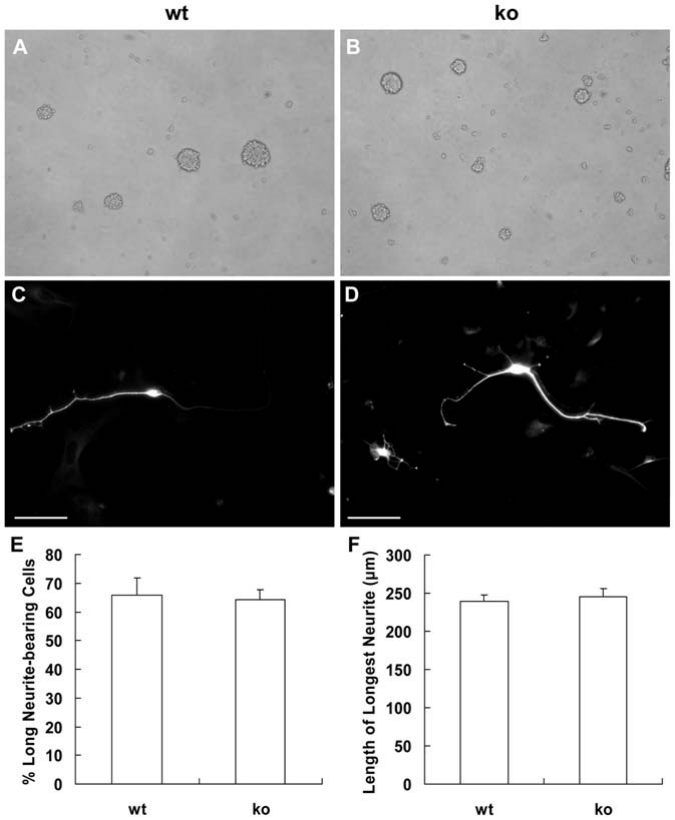
Target deletion of palladin does not impair neurite extension of neural stem cell-derived neurons. Neural stem cells (NSCs) are expanded as neurospheres *in vitro* (3DIV, A, B; magnification, 100×). Fluorescence immunostaining of Tuj1 in wild-type (wt, C) and palladin-deficient (ko, D) differentiated NSCs is shown (Scale bar = 100 µm). The Tuj1 positive cells are termed neurons. Quantification of neurite outgrowth based upon the Tuj1 positive cells described above. Cells with neurites of longer than two soma diameters were scored as long neurite-bearing cells. The percentage of long neurite-bearing cells is not significantly decreased in palladin-deficient NSC-derived neurons (E). The length of the longest neurite is not reduced in palladin-deficient NSC-derived neurons (F). Results are expressed as mean±SEM (n = 3).

The initial sprouting of neurites followed by elongation of axons and dendrites are principal morphological characteristics of neuronal differentiation. We therefore investigated the neurite morphogenesis in neural stem cell (NSC)-derived neurons. Neural stem cells from embryonic brain can be expanded *in vitro* as neurospheres in the presence of epidermal growth factor (EGF) and fibroblast growth factor-2 (FGF-2) (Figure 5A and B). Neurospheres can differentiate into astrocytes, oligodendrocytes and neurons [16]. These neurons can be identified by Tuj1 immunostaining (Figure 5C and D). The percentage of neurons with neurites longer than two soma diameters and the length of the longest neurite were parameters both measured on neurons found in fields of comparable cell density. The percentage of neurons with neurites longer than two soma diameters was 65.8±6.0% in wild-type NSC-derived neurons, and the percentage was 64.2±3.6% in palladin-deficient NSC-derived neurons (*P* = 0.824042). The length of the longest neurite of wild-type NSC-derived neurons was 239.9±7.7 µm, and that of palladin-deficient NSC-derived neurons was 244.8±11.2 µm (*P* = 0.734257). These findings demonstrate that palladin is unnecessary for normal neurite outgrowth in NSC-derived neurons.

We further observed the morphology of neurons derived from wild-type and palladin-deficient ES cells. To differentiate ES cells into neurons spontaneously *in vitro*, undifferentiated ES cells were cultivated as embryonic bodies (EBs) in hanging drops (500 cells/drop) for 2 days and in suspension for 1 day before being plated onto gelatin-coated 24-well tissue culture plates at one aggregate per well (Supplementary Figure S2A and B). Four weeks after plating, cells were fixed for Tuj1 immunostaining to identify the neurons and view the neuronal processes. In comparison with wild-type ESC-derived neurons, palladin-deficient ESC-derived neurons showed normal neurite extension as well (Supplementary Figure S2C–F).

## Discussion

Although palladin's molecular associations and *in vitro* functions have been described and knockdown of palladin expression by antisense constructs in cultured neurons and neuroblastoma cells have been shown to cause failure of neurite extension [10], palladin's *in vivo* role in the nervous system has not yet been explored. In this study, we have investigated the role of palladin in neurite extension *in vivo* and *in vitro*. Our results suggest that palladin is not essential for normal neurite outgrowth and elongation.

Many lines of evidence indicate that palladin plays a critical role in organizing the actin arrays required for normal cell adhesion and motility [8]. Using the palladin knockout mouse embryos and fibroblasts cultured from palladin knockout embryos, we confirmed this conclusion. The NTD and body wall defect of palladin-deficient embryos prove that palladin is required for normal cell motility and adhesion during embryogenesis [4]. In addition, palladin-deficient fibroblasts have shown defects in cell motility, adhesion, integrin expression, and actin organization [17]. Nevertheless, the neurite extension, which was expected to be stunted, was found to be normal both in palladin-deficient mouse embryos and in neurons derived from palladin-deficient embryos. Whole-mount immunostaing of NF clearly demonstrates that axon growth and morphology of DRG neurons innervating the limbs are normal in palladin-deficient embryos. In the forebrain and midbrain of mouse embryos, however, palladin deficiency causes failure of neural tube closure and eversion of neuroepithelium followed by perturbed innervations. Nevertheless, neurites do show extensive growth. This phenomenon indicates that palladin deficiency impairs cell movement and cell adhesion of neuroepithelium during neurulation, but does not impair neurite outgrowth and extension of neurons in the neuroepithelium. In addition, quantitative *in vitro* studies performed on primary cortical neurons confirm this conclusion.

To further understand the role of palladin during the development of the nervous system, we investigated the neuronal differentiation of palladin-deficient NSCs. Palladin-deficient NSCs are able to proliferate as neurospheres and to differentiate into neurons (Tuj1-positive), oligodendrocytes (O4-positive) and astrocytes (GFAP-positive) as wild-type NSCs. Moreover, the palladin deficiency does not change the percentage of neurons, oliogodendrocytes and astrocytes significantly after differentiation (data not shown). In addition, we performed neurite outgrowth assays on NSC derived-neurons, and demonstrated through quantitative analysis that loss of palladin does not inhibit neurite outgrowth. To fully understand the function of palladin, we also generated palladin-deficient ES cell lines. While examining the pluripotency of ES cells *in vitro*, we observed the morphology of neurons derived from ES cells. Preliminary observation indicates that the palladin-deficient ESC-derived neurons are able to extend neurites as long as wild-type ESC-derived neurons (Supplementary Figure S2).

During the development of the cerebral cortex, neurogenesis is ongoing in the compact ventricular zone (VZ) in the early phase, after which the post-mitotic neurons migrate to the marginal zone (MZ) [16], [18]. Accordingly, we have taken note of the cortical neuron distribution in the E13.5 cerebral cortex. In the wild-type cerebral cortex, neurons are located in the MZ. Though the palladin-deficient cerebral cortex fails to form cephalic ventricle structure and gives rise to the exposure of VZ to the outside, the cortical neurons are still able to migrate to the MZ (Supplementary Figure 1). It is likely that palladin deficiency does not impair the cortical neuron migration at an early developmental stage of the cerebral cortex. However, this assumption requires further study.

In our mouse model, all the 200 kd, 140 kd, 90–92 kd and 50 kd palladin isoforms are missing. These isoforms share the common C-terminus and the proteins have been confirmed based on our data and others [11]–[13]. Our data implicate that these palladin isoforms detected are dispensable for neurite outgrowth in mice. However, even more palladin isoforms are predicted according to the NIH AceView database reports, including the N-terminal isoforms do not share the common C-terminus [12]. For this reason, whether the N-terminal isoforms exist in the mouse nervous system and play a role in the neurite outgrowth still requires further investigations.

Palladin belongs to a family of actin-associated scaffolds including the Z-disc proteins myopalladin and myotilin. Myopalladin's expression is highly restricted to cardiac and skeletal muscle, while myotilin's expression is highest in skeletal muscle and moderate in heart and peripheral nerves. Though myotilin is highly expressed in the peripheral nervous system in mouse embryos during development, no report suggests that myotilin plays a role during neurite growth [19]. For this reason, further study is needed to determine whether myotilin can functionally compensate for loss of palladin during neurite growth.

We also try to understand the apparent discrepancy between the previous study and ours. In the previous study, down-regulation of palladin in cultured neurons and neuroblastoma cells was achieved by using the vector expressing the palladin antisence fragment which is derived from the C-terminus of palladin (AF205078). The acute down-regulation of the expression of palladin isoforms sharing the C-terminus of palladin led to inhibited neurite outgrowth in the cultured cells. Comparing to previous study, our study is based on the genetic modified mice, in which part of the palladin C-terminus was deleted. It is possible that the function of palladin in mouse nervous system could be compensated by other unknown proteins *in vivo* during the development of mouse embryogenesis.

In summary, we report that palladin is not essential for normal neurite outgrowth. However, palladin function in growth cone behavior requires further exploration. It is reasonable to generate palladin conditional knockout mice in order to study palladin function in mice after embryogenesis. Under these conditions, more classic experiments, for example, assays on axon guidance and axonal connectivity, can be performed to help build a fuller understanding of the function of palladin in the nervous system.

## Materials and Methods

### Animals

Mice containing a heterozygous deletion for palladin were maintained on 129SvJ background. Genotypes of adult mice, embryos and ES cells were analyzed as described previously [4]. All the procedures described were approved by the Animal Use and Care Committee of Shanghai Jiao Tong University School of Medicine.

### Reverse Transcription PCR

Total RNA was isolated from tissue or cells using the TRIzol reagent according to the manufacturer's protocol (Invitrogen, Carlsbad, CA). DNase I-treated (Promega, Madison, WI) total RNA was reverse transcribed to cDNA using AMV reverse transcriptase (Takara, Otsu, Japan). Different palladin isoforms were amplified using specific primers as described previously [11]. The common C-terminus of the palladin isoforms was amplified using the primer as described in [4].

### Western Blotting

Frozen tissue was homogenized in RIPA buffer containing Protease Inhibitor Cocktail (Sigma, St. Louis, MO) and PMSF with a homogenizer and a sonicator at 4°C. After centrifuging at 10,000×g for 10minutes at 4°C, the supernatant protein was quantified by the Bio-Rad DC Protein Assay (Hercules, CA). Tissue homogenates (40 µg) were separated by SDS-PAGE and transferred to nitrocellulose membrane. Antibodies of palladin (PTG, Chicago, USA), and α-actin (Sigma) were used as probes. Immunoblots were detected using the Odyssey® Infrared Imaging System (LI-COR Biotechnology, Nebraska, USA) referred to the manufacturer's protocols.

### Immunohistochemistry of whole mount mouse embryos

Peripheral innervation was visualized using antibodies against neurofilament (NF-M) (Santa-cruz Biotechnology, CA). All procedures were performed as previously described [20]. Photographs of whole-mounts were taken using a dissecting microscope equipped with an ECD capturer.

### Cortical neuron culture and transfection

Cells dissected from E14.5 mouse forebrains were mechanically dissociated and digested with 0.125% trypsin for 10 min at 37°C, followed by trituration with pipettes in the plating medium (DMEM with 10% FBS and 10% F12, Gibco, Carlsbad, CA). The single cell suspensions were plated onto coverslips coated with poly-D-lysine (100 µg/ml, Sigma) at a density of 5×10^4^/cm^2^. After culturing for 30 min, media was changed and cells were cultured in neuronal culture media (neurobasal media containing 1% glutamate and 2% B27, Gibco). Primary cortical neurons in culture were transfected with green fluorescent protein (GFP) plasmid alone (Clontech, Palo Alto, CA) 3 hrs after they were plated using Lipofectamine 2000 reagent (Invitrogen) as previously described [10].

### Neurosphere culture and differentiation

Cells dissected from E13.5 mouse forebrains were mechanically dissociated and placed into 35 cm^2^ flasks (Corning, NY, USA) at 0.5–1×10^5^ cells/ml with N-2 plus medium. N-2 plus medium consists of DMEM/F-12 (1∶1, Gibco) and N-2 plus media supplement (1∶100, R&D Systems, Minneapolis, USA). Media were supplemented with 10 ng/ml FGF-2 and 10 ng/ml EGF (R&D Systems) every 2–3 days. After 6 days in culture, neurospheres were plated onto 12 mm coverslips coated with poly-D-lysine (100 µg/ml, Sigma) and placed into 6-well plates. Subsequently, 2 ml culture medium consisting of DMEM and 2% B27 supplement (Gibco) were added to each well. After 3 days of adhesion, the majority of cells had migrated out of the sphere. These cells were then fixed for immunochemistry.

### Palladin-deficient mouse ES cell derivation and culture

Mouse embryonic fibroblast (MEF) cells were derived from E12.5 mouse embryos. Supportive irradiated MEFs of passage 3–4 were used as feeder layers during mouse ES cell derivation. In order to generate wild-type and palladin-deficient ES cells, heterogenous mice were mated and all blastocysts were flushed from the uterus at E3.5. Zona pellucida was removed by a mechanical procedure, and each individual whole embryo was placed onto a feeder layer-coated 4-well plate with mouse ES medium (DMEM with 10% FBS (Gibco), 0.1 mM mercaptothanol, 2 mM L-glutamine, 1000 units/ml murine leukemia inhibitor factor (Chemicon) and 2 µm GSK-3 inhibitor). Four to five days later, expanded typical ICM outgrowths were picked up carefully and transferred onto fresh feeder layers. When approximately 100 cells were found in each clone, trypsin was used to disaggregate the ES colonies in the following passage. DNA for genotyping was extracted from ES cells growing upon gelatinized plates (without MEFs).

### Immunochemistry

The cells on coverslips were fixed with 4% PFA in phosphate-buffered saline (PBS) for 20 min at room temperature. After three rinses in PBS (10 min each), the cells were incubated with 0.1% TritonX-100 in PBS for 10 min at room temperature, and then with 10% normal goat serum for 1 hr at 37°C. Following incubation with Tuj-1 antibodies (Promega, Madison, WI) overnight at 4°C, cells were rinsed three times in PBS and incubated with secondary antibodies in the dark. Cells were photographed with a microscope equipped with an ECD capturer.

### Histology and immunohistochemistry

Tissues were fixed in 4% PFA, soaked in 30% sucrose in PBS overnight at 4°C, embedded in paraffin and sectioned. Sections were stained with hematoxylin and eosin by standard procedures or immunostained with Map-2 antibody (Santa-cruz Biotechnology) as described above, then photographed with a microscope equipped with an ECD capturer.

### Length of the longest neurite

Photomicrographs were imported into the LSM 5 Image Examiner software (Carl Zeiss Inc, US), where the length of the longest neurite was measured on at least 20 neurons per sample. At least three independent experiments were performed on cells derived from different mouse embryos.

### Statistical analysis

Data are expressed as mean±SEM. Statistical significance between any 2 groups was determined by the 2-tailed Student *t* test. *P* values less than 0.05 were considered significant.

## Supporting Information

Figure S1 HE staining (coronal section) of E13.5 mouse embryo forebrain. The palladin-deficient (ko) neuroepithelium (B) cannot fuse to form a normal cephalic ventricle structure as would be observed in wild-type embryos (wt, A). As a result, the ventricular zone (VZ) is exposed to the outside in ko embryos (D) instead of the marginal zone (MZ, C). However, fluorescence immunostaining of Map-2 (coronal section, E and F) shows that the neurons do locate in the MZ in ko embryos.
(3.60 MB TIF)

Figure S2 Target deletion of palladin does not inhibit neurite extension of embryonic stem cell-derived neurons. Palladin-deficient (ko) embryonic stem (ES) cells can differentiate into neurons, and these ES cell-derived neurons are able to extend neurites as long as those of wild-type (wt) cells. ES cells were cultivated to form embryonic bodies (3DIV, A, B). Fluorescence immunostaining of Tuj1 in vitro differentiated wild-type (C, E) and palladin-deficient (D, F) ES cells is shown (31DIV; magnification, 100×).
(3.88 MB TIF)
