# Supplementary figures and images for: Target Deletion of the Cytoskeleton-Associated Protein Palladin Does Not Impair Neurite Outgrowth in Mice
Source: PLoS One. 2009 Sep 4;4(9):e6916. doi: 10.1371/journal.pone.0006916 (PMC2731857; doi:10.1371/journal.pone.0006916)

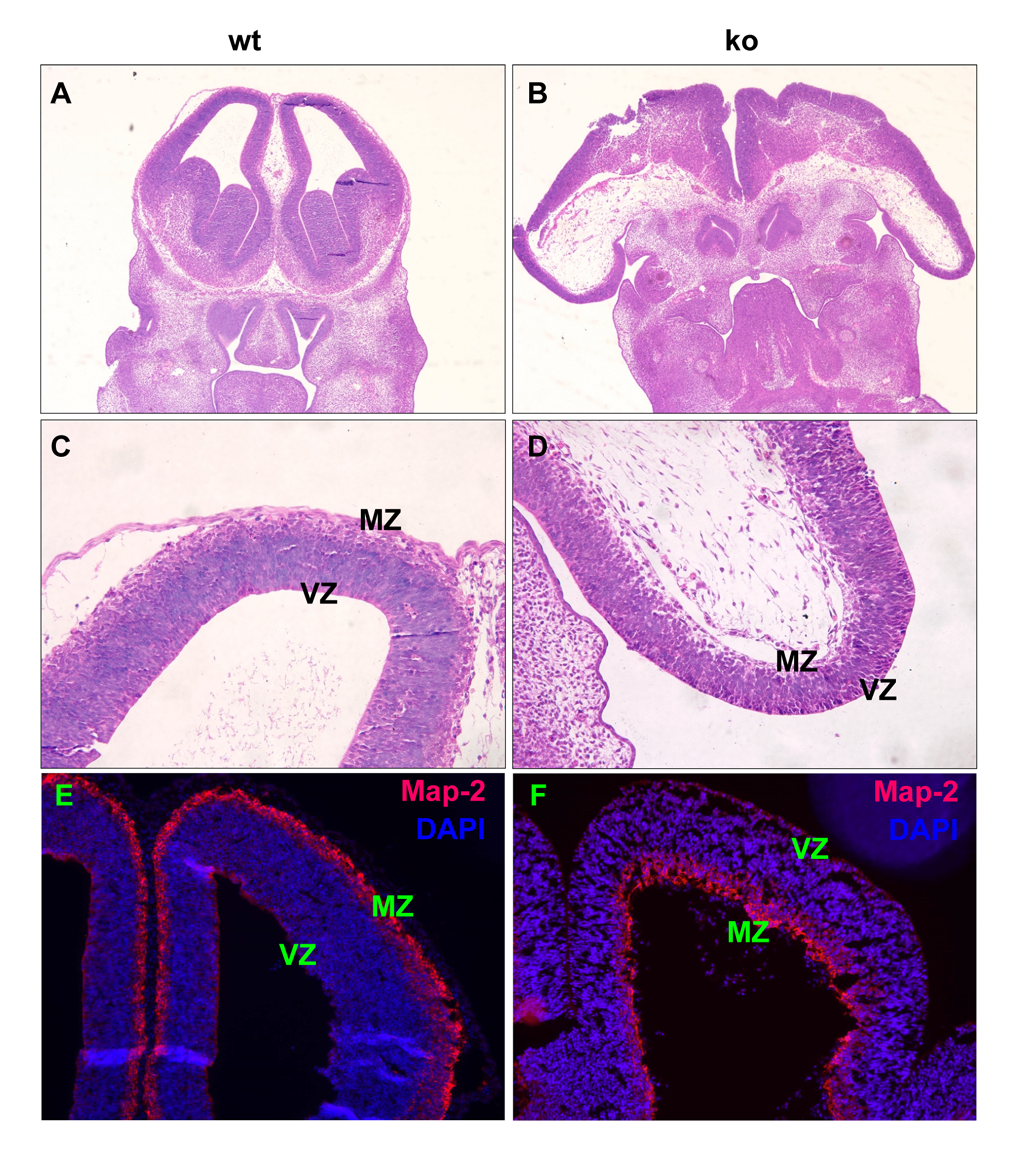

Supplement: Figure S1 — HE staining (coronal section) of E13.5 mouse embryo forebrain. The palladin-deficient (ko) neuroepithelium (B) cannot fuse to form a normal cephalic ventricle structure as would be observed in wild-type embryos (wt, A). As a result, the ventricular zone (VZ) is exposed to the outside in ko embryos (D) instead of the marginal zone (MZ, C). However, fluorescence immunostaining of Map-2 (coronal section, E and F) shows that the neurons do locate in the MZ in ko embryos. (3.60 MB TIF) [file pone.0006916.s001.tif]

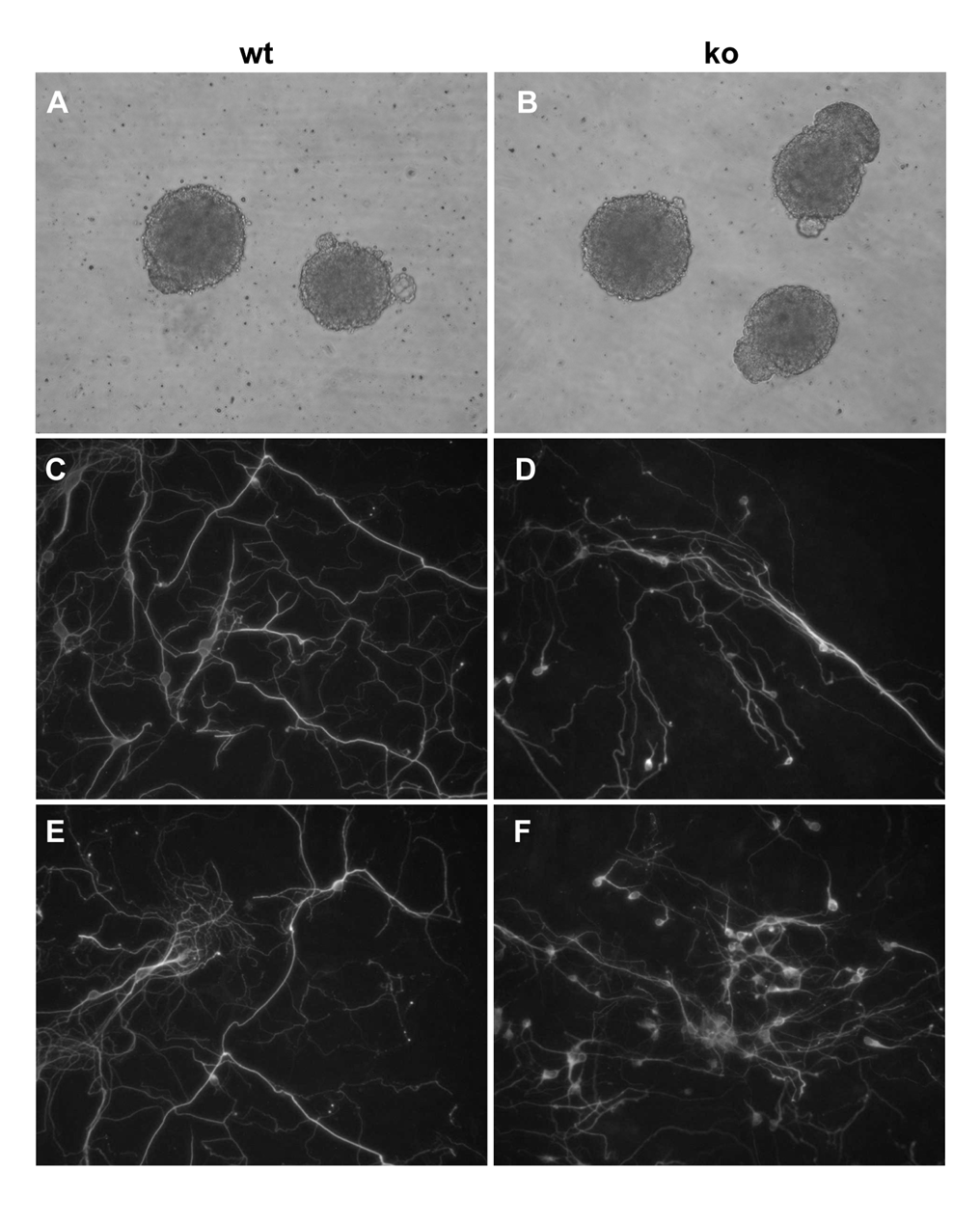

Supplement: Figure S2 — Target deletion of palladin does not inhibit neurite extension of embryonic stem cell-derived neurons. Palladin-deficient (ko) embryonic stem (ES) cells can differentiate into neurons, and these ES cell-derived neurons are able to extend neurites as long as those of wild-type (wt) cells. ES cells were cultivated to form embryonic bodies (3DIV, A, B). Fluorescence immunostaining of Tuj1 in vitro differentiated wild-type (C, E) and palladin-deficient (D, F) ES cells is shown (31DIV; magnification, 100×). (3.88 MB TIF) [file pone.0006916.s002.tif]
